# Daemonic Ergotropy: Generalised Measurements and Multipartite Settings

**DOI:** 10.3390/e21080771

**Published:** 2019-08-07

**Authors:** Fabian Bernards, Matthias Kleinmann, Otfried Gühne, Mauro Paternostro

**Affiliations:** 1 Naturwissenschaftlich-Technische Fakultät, Universität Siegen, Walter-Flex-Straße 3, 57068 Siegen, Germany; 2 School of Mathematics and Physics, Queen’s University, Belfast BT7 1NN, UK

**Keywords:** ergotropy, quantum correlations, information thermodynamics

## Abstract

Recently, the concept of daemonic ergotropy has been introduced to quantify the maximum energy that can be obtained from a quantum system through an ancilla-assisted work extraction protocol based on information gain via projective measurements [G. Francica et al., npj Quant. Inf. **3**, 12 (2018)]. We prove that quantum correlations are not advantageous over classical correlations if projective measurements are considered. We go beyond the limitations of the original definition to include generalised measurements and provide an example in which this allows for a higher daemonic ergotropy. Moreover, we propose a see-saw algorithm to find a measurement that attains the maximum work extraction. Finally, we provide a multipartite generalisation of daemonic ergotropy that pinpoints the influence of multipartite quantum correlations, and study it for multipartite entangled and classical states.

## 1. Introduction

In the rapidly evolving research arena embodied by the thermodynamics of quantum systems, the resource-role of quantum features in work-extraction protocols is one of the most interesting and pressing open questions [1,2,3,4]. Quantum coherences are claimed to be responsible for the extraction of work from a single heat bath [5] and the enhanced performance of quantum engines [6]. Weakly driven quantum heat engines are known to exhibit enhanced power outputs with respect to their classical (stochastic) versions [7]. Quantum information-assisted schemes for energy extraction have been put forward and shown to be potentially able to achieve significant efficiencies [8,9,10,11,12,13]. However, controversies in the usefulness of quantum correlations and coherences in schemes for the extraction of work from quantum systems have also been discussed [14,15,16,17]. While a full physical understanding of these issues is still far from being acquired, theoretical progress in this direction will be key to the design and implementation of informed experimental proof-of-principle experiments and thus the consolidation of a quantum approach to the thermodynamics of microscopic systems.

Recently, a simple ancilla-assisted work-extraction protocol has been proposed that is able to pinpoint the crucial role that quantum measurements have in the performance of a quantum work-extraction game. This protocol also highlighted important implications arising from the availability of quantum correlations between the work medium and the ancilla [18]. The scheme provided a link between enhanced work extraction capabilities and quantum entanglement between ancilla and work medium, suggesting the possibility to exploit entanglement as a resource.

In this work we show that although this link exists for pure states, quantum correlations and work extraction capabilities are unrelated if mixed states are considered. However, the scheme in Reference [18] relied on a set of very stringent assumptions, which leave room to further investigations aimed at clarifying the potential benefits of exploiting quantum resources. Here, we critically investigate the protocol in Reference [18], and extend it in various directions. First, we address the class of measurements that ensure the enhancement of the work-extraction performance. We provide an example in which generalised measurements allow for more extracted energy than projective measurements do. The search for the right generalised measurement poses serious computational challenges that we solve by proposing a constructive see-saw algorithm that is able to identify the most effective measurement for a given state of the work medium and ancilla, and an assigned Hamiltonian of the former. We then address the issue embodied by the interplay between information gathered via optimal measurements and quantum correlations shared between work medium and ancilla. We show that, depending on the nature of the optimal measurement, quantum correlations may become entirely inessential for the enhancement of work extraction. Finally, we open the investigation to multipartite settings by addressing the case of multiple work media and ancillas, showing that the structure of correlation-sharing among the various parties of such a system is key in the performance of our work-extraction protocol.

Our results contribute to the ongoing research for the ultimate resources to be exploited to draw an effective and useful framework for quantum enhanced thermodynamical processes. While clarifying a number of important points, our work opens up new avenues of investigation that will be crucial for the design of unambiguous experimental validations.

## 2. Notation and Concepts

The maximal energy decrease of a given state $\varrho^{S}$ with respect to a reference Hamiltonian *H* undergoing an arbitrary unitary evolution *U* is its ergotropy [19]

(1) $$\begin{array}{c} W\left(\varrho^{S},H\right)=\mathrm{Tr}\left[\varrho^{S}H\right]-\min_{U}\mathrm{Tr}\left[U\varrho^{S}U^{†}H\right]. \end{array}$$

This is interpreted as the maximal amount of work that can be extracted from a system prepared in state $\varrho^{S}$ by the means of a unitary protocol [19]. Given some state in its spectral decomposition $\varrho^{S}\;=\;\sum_{k}r_{k}\;|r_{k}\rangle \langle r_{k}|$ with $r_{k+1}\leq r_{k}$ and a Hamiltonian $H=\sum_{k}\epsilon_{k}|\epsilon_{k}\rangle \langle \epsilon_{k}|$ with $\epsilon_{k+1}\geq \epsilon_{k}$ the optimal unitary is $U\;=\;\sum_{k}|\epsilon_{k}\rangle \langle r_{k}|$ [19]. This is a direct consequence of the von Neumann trace inequality [20]. It states that $\mathrm{tr}\left[AB\right]\;\leq \;\sum_{i}a_{i}b_{i}$, where $a_{i}\;\left(b_{i}\right)$ are the eigenvalues of A(B) and $a_{i+1}\geq a_{i},\;b_{i+1}\;\geq \;b_{i}$. Choosing $A=-U\varrho^{S}U†$ and B=H and writing $\max_{U}\mathrm{tr}\left[-U\varrho^{S}U†H\right]=-\min_{U}\mathrm{tr}\left[U\varrho^{S}U†H\right]$ then shows that the bound given by the von Neumann trace inequality is achieved with the unitary stated above.

**Figure 1:**
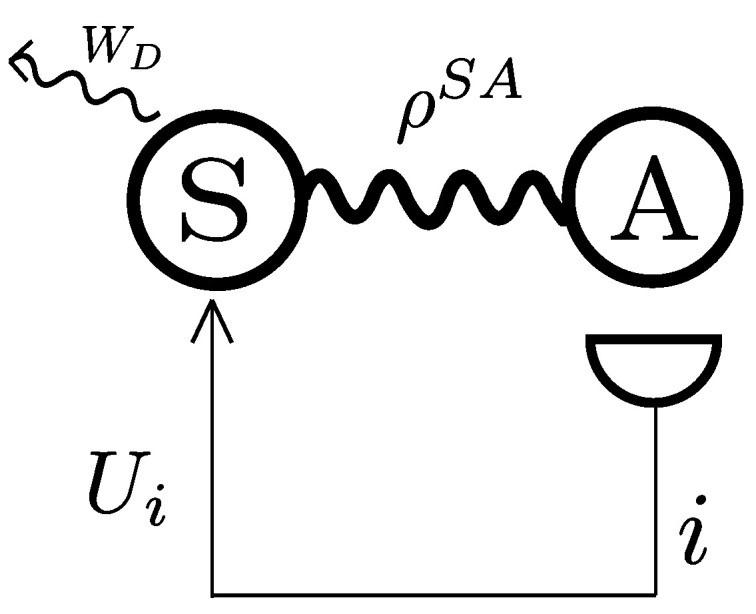
Illustration of daemonic ergotropy. A system *S* is coupled to an ancilla *A*. A measurement is performed on the latter and depending on the outcome *i* different unitaries can be applied to *S* in order to extract work. The maximal amount of extractable work using this protocol is the daemonic ergotropy.

In Reference [18], an ancilla-assisted protocol allowed for enhanced work extraction by making use of a process of information inference. The fundamental building blocks of the protocol are embodied by the joint state of a work medium *S* and an ancilla *A*, and a projective measurement *M* performed on the latter (cf. Figure 1). The information gathered through these measurements is then used to determine a unitary transformation to be applied to *S* to extract as much work as possible.

This work, which is dubbed daemonic ergotropy, is given by

(2) $$W_{D}\left(\varrho^{SA},H,M\right)=\mathrm{Tr}\left[\varrho^{S}H\right]-\sum_{i}\min_{U_{i}}\mathrm{Tr}\left(\gamma_{i}^{S}{\tilde{H}}_{i}\right),$$

where ${\tilde{H}}_{i}=U_{i}^{†}HU_{i}$, $M=\{\Pi_{j}\}$ is a projective measurement, and $\gamma_{i}^{S}={\mathrm{Tr}}_{A}\left[\varrho^{SA}\left(I^{S}⊗\Pi_{i}^{A}\right)\right]$ is the unnormalised conditional state of *S* corresponding to the *i*th measurement outcome. The daemonic ergotropy can be written in a more compact way using the ergotropy, namely

(3) $$W_{D}\left(\varrho^{SA},H,M\right)=\sum_{i}W\left(\gamma_{i}^{S},H\right).$$

For a pure state, any projective measurement *M* with $\Pi_{i}$ rank-one projectors maximises the daemonic ergotropy. In fact, the conditional states $\gamma_{i}^{S}$ are then pure and it is always possible to find a unitary—specific to every conditional state—that maps it to the ground state of the Hamiltonian, thus lowering as much as possible the energy of the system and extracting the maximum amount of work [18].

The difference between maximal daemonic ergotropy and ergotropy is called daemonic gain [18], and is formalised as

(4) $$\begin{array}{c} \delta W\left(\varrho^{SA},H\right)=\max_{M}W_{D}\left(\varrho^{SA},H,M\right)-W\left(\varrho^{S},H\right). \end{array}$$

If $\varrho^{SA}$ is a pure product state, $\varrho^{S}$ is pure. Thus, no measurement on the ancilla is required for optimal work extraction, since in this case there is a unitary that maps $\varrho^{S}$ to the ground state of the Hamiltonian. Consequently, the daemonic ergotropy coincides with the ergotropy in this case and there is no daemonic gain.

The definitions provided above pinpoint the key role of the measurement step in such an ancilla-assisted extraction protocol. In particular, the assumption of projective measurements performed on *A* appears to be too restrictive. It is thus plausible to wonder if better performances of the daemonic work-extraction scheme are possible when enlarging the range of possible measurements on the ancilla to generalised quantum measurements.

## 3. Non-Optimality of Projective Measurements for Daemonic Ergotropy

We now address such a scenario and provide an example where more energy can be extracted from *S* when generalised measurements are performed. To this end, we will employ the formalism of positive operator valued measures (POVMs) [21]. In the case of a finite set of outcomes {i}, a POVM is a map that assigns a positive semidefinite operator $E_{i}$—dubbed as effect—to each outcome *i*, such that $\sum_{i}E_{i}=I$. As with projective measurements, the probabilities for the outcomes are obtained as $p_{i}=\mathrm{Tr}\left(E_{i}\varrho \right)$. However, the effects $E_{i}$ of a POVM need not be projectors.

Let us consider now a three-level system *S* and a two-level ancilla *A* prepared in the joint state

(5) $$\begin{array}{c} \varrho^{SA}=\frac{1}{3}\sum_{j=0}^{2}\left|j\rangle \langle j\right|⊗\Pi \left(\frac{2\pi j}{3},0\right) \end{array}$$

with projectors

(6) $$\Pi \left(\alpha ,\beta \right)=\frac{1}{2}\left\{I+\cos\left(\alpha \right)\sigma_{z}+\sin\left(\alpha \right)\left[\cos\left(\beta \right)\sigma_{x}-\sin\left(\beta \right)\sigma_{y}\right]\right\}.$$

Here (α,β) are angles in the single-qubit Bloch sphere. We assume a reference Hamiltonian $H=\sum_{j}\epsilon_{j}\left|j\rangle \langle j\right|$ with energy eigenvalues $\epsilon_{j}$ arranged in increasing order. If only projective measurements *M* are allowed on the state of the ancilla, the maximum daemonic ergotropy achieved upon optimizing over the measurement strategy is

(7) $$\begin{array}{c} \max_{M}W_{D}\left(\varrho^{SA},H,M\right)=W\left(\varrho^{S},H\right)+\frac{\epsilon_{2}-\epsilon_{0}}{2\sqrt{3}}. \end{array}$$

Details on this result are presented in Appendix A. However, if generalised measurements are permitted, one may choose the POVM with effects $E_{j}=\frac{2}{3}\Pi \left(2\pi j/3,0\right)$ to yield a daemonic ergotropy of

(8) $$\begin{array}{c} W_{D}\left(\varrho^{SA},H,\left\{E_{i}\right\}\right)=W\left(\varrho^{S},H\right)+\frac{1}{6}\left(\epsilon_{1}+\epsilon_{2}-2\epsilon_{0}\right). \end{array}$$

**Figure 2:**
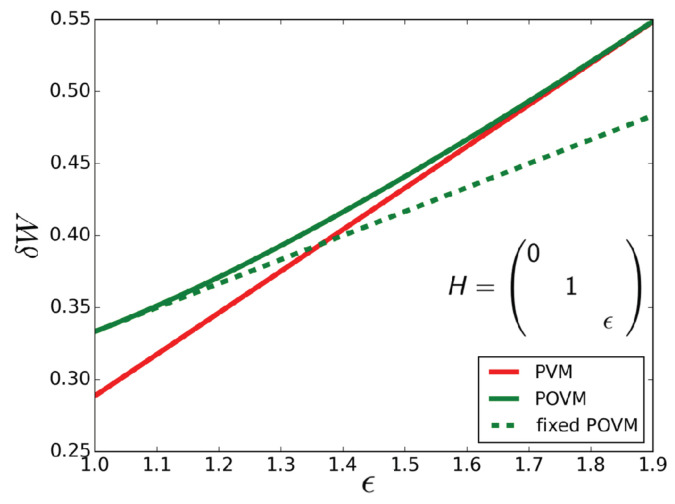
Daemonic gain δW as a function of the value of the highest energy level of the Hamiltonian *H* (in units of $\epsilon_{1})$ for the state $\varrho^{SA}$ given in Equation (5). Here $\epsilon =\epsilon_{2}/\epsilon_{1}$. We compare the performance under the optimal r projective measurements (PVM) and positive operator valued measures (POVM). The latter was found numerically using the see-saw algorithm proposed here. The former is determined analytically as discussed in Appendix A. The dashed line is obtained as the daemonic gain δW for the fixed POVM with effects $E_{j}=\frac{2}{3}\Pi \left(2\pi j/3,0\right)$.

This can exceed the maximum daemonic ergotropy achieved through projective measurements. For instance, we can assume to have shifted energy so that $\epsilon_{0}=0$. Under such conditions, we would have $W_{D}\left(\varrho^{SA},H,\left\{E_{i}\right\}\right)>\max_{M}W_{D}\left(\varrho^{SA},H,M\right)$ for $\left(\sqrt{3}-1\right)\epsilon_{2}<\epsilon_{1}\leq \epsilon_{2}$. Figure 2 shows the daemonic gain δW corresponding to the example above as a function of the value of the highest energy level of the Hamiltonian for projective measurements (PVMs) and POVMs. While in this example the optimal projective measurement does not depend on the Hamiltonian, the optimal POVM does. Therefore, the daemonic gain grows linearly with the value of the highest energy value, as long as only projective measurements are taken into account. For comparison, the daemonic gain that can be achieved with the previously discussed POVM ${\left(\frac{2}{3}\Pi \left(2\pi j/3,0\right)\right)}_{j}$ is plotted as a dashed line.

## 4. Construction of Optimal POVMs

Having provided a useful example, we now move to address the problem of identifying the ideal POVM for optimal daemonic ergotropy. The following Lemma is instrumental to the achievement of our goal:

**Lemma** **1.** *The ergotropy is a sublinear function in its first argument, which refers to the state. That is, for any $\gamma =\gamma_{1}+\gamma_{2}$*

(9) $$\begin{array}{c} W\left(\gamma ,H\right)\leq \sum_{i=1,2}W\left(\gamma_{i},H\right) \end{array}$$

*and*

(10) $$\begin{array}{c} W(\lambda \gamma ,H)=\lambda W(\gamma ,H) \end{array}$$

*for any λ≥0. As ergotropy is symmetric under the exchange of its first and the second argument, it is also sublinear in the Hamiltonian.*

**Proof.**  The second equation holds trivially, which justifies our use of unnormalised states. We obtain the first inequality as follows

(11) $$\begin{array}{cc} W(\gamma ,H) & =\mathrm{Tr}\left(\gamma H\right)-\min_{U}\mathrm{Tr}\left[U\gamma U^{†}H\right]\\ & \leq \sum_{j=1,2}\left[\mathrm{Tr}\left(\gamma_{j}H\right)-\min_{U}\mathrm{Tr}\left(U\gamma_{j}U^{†}H\right)\right]\\ & =\sum_{j=1,2}W\left(\gamma_{j},H\right). \end{array}$$

 □

Note that sublinearity implies convexity, i.e., $W\left[\lambda \gamma_{1}+\left(1-\lambda \right)\gamma_{2},H\right]\leq \lambda W\left(\gamma_{1},H\right)+\left(1-\lambda \right)W\left(\gamma_{2},H\right)$. This result allows us to state the following corollary:

**Corollary** **2.** *The daemonic ergotropy*

(12) $$\begin{array}{cc} W_{D}\left(\varrho^{SA},H,M\right)= & \sum_{i}W\left(\gamma_{i}^{S},H\right)\geq W\left(\sum_{i}\gamma_{i}^{S},H\right)=W\left(\varrho^{S},H\right) \end{array}$$

*is larger or equal to ergotropy. Equality holds for the trivial measurement, with the identity as only effect.*

This claim has already been proven in a different way in Reference [18]. A second interesting consequence of the sublinearity of ergotropy is stated in the following lemma:

**Lemma** **3.** *Daemonic ergotropy is a convex function of its third argument, which pertains to the measurement strategy.*

**Proof.**  Let us consider a mixed measurement strategy Q=λM+(1−λ)N with 0≤λ≤1, and the corresponding daemonic ergotropy. We have

(13) $$\begin{array}{cc} W_{D}\left[\varrho^{SA},H,Q\right] & \leq \lambda \sum_{i}W\left[{\mathrm{Tr}}_{A}\left(\varrho^{SA}I⊗M_{i}\right),H\right]+\left(1-\lambda \right)\sum_{i}W\left[{\mathrm{Tr}}_{A}\left(\varrho^{SA}I⊗N_{i}\right),H\right]\\ & =\lambda W_{D}\left(\varrho^{SA},H,M\right)+\left(1-\lambda \right)W_{D}\left(\varrho^{SA},H,N\right). \end{array}$$

 □

We complete our formal analysis that precedes the presentation of an algorithm for the identification of the optimal POVM with the following theorem.

**Theorem** **4.** *For any state $\varrho^{SA}$ and any POVM M, one can find a POVM $\tilde{M}$ with at most $d^{2}$ effects, where d is the dimension of the ancilla, such that*

(14) $$\begin{array}{c} W_{D}\left(\varrho^{SA},H,M\right)=W_{D}\left(\varrho^{SA},H,\tilde{M}\right). \end{array}$$

**Proof.**  The set of POVMs on a *d* dimensional system is convex and it has been shown that the extremal points of this set are POVMs with at most $d^{2}$ effects [22]. A convex function that is defined on a convex domain takes its maximum on an extremal point. Therefore, there is an extremal POVM *E* with *n* outcomes, $1\leq n\leq d^{2}$, that exhibits a daemonic ergotropy that is larger than or equal to the daemonic ergotropy for *M*. If equality holds, we choose $\tilde{M}=E$. Otherwise, there is a mixture $\tilde{M}=\lambda E+\left(1-\lambda \right)I$ between *E* and a trivial random measurement *I* with *n* outcomes and effects $I_{i}=I/n$ that meets the requirement, since $W_{D}\left(\varrho^{SA},H,I\right)=W\left(\varrho^{S},H\right)\leq W\left(\varrho^{SA},H,M\right)$. □

We are now in the position to present an algorithm for the search of the optimal measurement. This task involves two parts (a) Finding the optimal measurement and (b) Finding the optimal unitaries to calculate the ergotropies of the conditional states. Assume a fixed measurement. Then, the conditional states are fixed and one can find the optimal unitaries as discussed in the introduction after Equation (1). On the other hand, if some $d^{2}$ unitaries $U_{i}$ are given, finding the optimal measurement $M={\left(E_{i}\right)}_{i}$ is a semidefinite program (SDP) [23]

(15) $$\begin{array}{cc} \min_{M} & \sum_{i}\mathrm{Tr}\left(\tau_{i}E_{i}\right)\\ s.t\; & \sum_{i}E_{i}=I\\ & E_{i}\geq 0 \end{array}$$

where $E_{i}$ are the effects associated with the POVM *M* and

(16) $$\begin{array}{c} \tau_{i}={\mathrm{Tr}}_{S}\left(\varrho^{SA}U_{i}^{†}HU_{i}\right). \end{array}$$

We thus propose the following see-saw Algorithm 1:

**Algorithm 1** Optimise POVM for daemonic ergotropy1: Choose *n* different unitaries $U_{i}$ and calculate $\tau_{i}$2: Solve the SDP above. This will yield a POVM *M*.3: Calculate the conditional states $\gamma_{i}^{S}$ for the POVM *M* and the optimal unitaries $U_{i}$.4: **repeat**▹ Iterate steps 2 and 35: **until** convergence.

We can restrict ourselves to $n=d^{2}$ different unitaries in the first step because of Theorem 4. Calculating the daemonic ergotropy after every round of the algorithm will yield a monotonically increasing sequence that is bounded from above because all involved operators are bounded and will therefore converge. In the case of the example discussed above, roughly 10 iterations are needed until the limit is reached within numerical precision. The sequence however sometimes converges to a local maximum that is strictly smaller than the maximal daemonic ergotropy. Besides observing this in practice, we also construct such a case in Appendix B.

## 5. The Role of Quantum Correlations

Notwithstanding the handiness of the algorithm built above, analytical solutions can be found in some physically relevant cases. The one most pertinent to the scopes of this work [18] is embodied by quantum-classical *S*-*A* states, i.e., states that can be cast in the form

(17) $$\begin{array}{c} \varrho_{qc}^{SA}=\sum_{j}\sigma_{j}^{S}⊗{\left|j\rangle \langle j\right|}^{A} \end{array}$$

with ${\{\;|j\rangle }^{A}\}$ a set of orthonormal vectors and $\sigma_{j}^{S}$ unnormalised states. This class of states has attracted attention from the community interested in the characterization of general quantum correlations, for it has only classical correlations, that is, it is not entangled and exhibits no quantum discord, if *A* is considered as the system the measurement being performed on [24,25,26,27]. For these states, we provide the following theorem. The proof is found in Appendix C.

**Theorem** **5.** *For a quantum-classical state $\varrho_{qc}^{SA}$, the maximum daemonic ergotropy is*

(18) $$\begin{array}{c} \max_{M}W_{D}\left(\varrho^{SA},H,M\right)=\sum_{j}W\left(\sigma_{j}^{S},H\right). \end{array}$$

*This value is achieved by performing the projective measurement with effects $P_{j}={\left|j\rangle \langle j\right|}^{A}$(j=1,…,d) on the ancilla A.*

This shows that, in the case of a quantum-classical state, we have an analytic form for the daemonic gain. To calculate it, we should diagonalise the reduced state $\varrho^{A}={\mathrm{Tr}}_{S}\left(\varrho^{SA}\right)$ of the ancilla. This yields a unitary to make the state block-diagonal. The individual blocks are then the optimal conditional states $\sigma_{j}^{S}$ that one needs in order to compute the daemonic gain.

The above result paves the way to an investigation on the role that quantum correlations play in the daemonic protocol for work extraction. This important question was already partially addressed in Reference [18], where a very close relation between daemonic gain and entanglement in pure *S*-*A* states was pointed out, while the link was shown to be looser for the case of mixed resource states.

Here, by using the results reported above, we shed further light on the link between daemonic gain and quantum correlations. Let us assume that, for a given resource state $\varrho^{SA}$, the optimal measurement for daemonic gain is projective, and call $P_{i}=\left|i\rangle \langle i\right|$ the corresponding projections, which can be chosen, without loss of generality, to be rank one. We write the resource state as

(19) $$\begin{array}{c} \varrho^{SA}=\sum_{ij}^{S}\sigma_{ij}^{S}⊗{\left|i\rangle \langle j\right|}^{A}, \end{array}$$

where the dyads ${\left|i\rangle \langle j\right|}^{A}$ are written in the basis defined by the optimal projectors $P_{i}$ above. We notice that all off-block-diagonal terms $\sigma_{ij}^{S}$ (with i≠j) do not contribute to the daemonic gain, which is thus the same as the one associated with the quantum-classical state

(20) $$\begin{array}{c} \varrho_{qc}^{SA}=\sum_{i}\sigma_{ii}^{S}⊗{\left|i\rangle \langle i\right|}^{A}. \end{array}$$

That this state is a quantum-classical state is obvious from the definition provided in Equation (17). This state can be produced by performing the optimal measurement and preparing a pure state on the ancilla accordingly. This procedure destroys all the quantum correlations, while the daemonic gain remains unchanged. Quantum correlations in the resource states are thus not useful, if the optimal measurement is projective. This is especially true if only projective measurements are considered from the start, which stresses the importance of considering generalised measurements, if one aims at investigating the impact entanglement may have on daemonic ergotropy.

However, we now show that, even if we allow for the use of arbitrary POVMs, the maximum daemonic gain for any given Hamiltonian can be achieved by classical-classical states, i.e., states whose parties share only classical correlations [26]. We do this by providing an upper bound on the daemonic gain. This bound is tight as it is achieved by a classical-classical state. Let us consider an explicit formula for daemonic gain, where we have inserted the definitions of ergotropy and daemonic ergotropy. We have

(21) $$\begin{array}{cc} \delta W(\varrho^{SA},H) & =\min_{U}\mathrm{Tr}\left(U\varrho^{S}U^{†}H\right)-\min_{\left(E_{k}\right)}\min_{U_{k}}\sum_{k}\mathrm{Tr}\left(U_{k}\varrho_{k}^{S}U_{k}^{†}H\right). \end{array}$$

Using von Neumann’s trace inequality, which reads $\mathrm{Tr}\left(AB\right)\leq \sum_{i}a_{i}b_{i}$ with $a_{i}\left(b_{i}\right)$ the eigenvalues of A (B) in increasing order, one easily finds that the first term never exceeds $\frac{1}{d_{S}}\mathrm{Tr}\left(H\right)$, where $d_{S}$ is the dimension of the Hilbert space of *S*. This value is attained if $\varrho^{S}$ is maximally mixed. The smallest value that the second term can take is $\epsilon_{0}$, the lowest energy eigenvalue. This is achieved for pure conditional states $\varrho_{k}^{S}$. Consequently

(22) $$\begin{array}{c} \delta W\left(\varrho^{SA},H\right)\leq \frac{1}{d_{S}}\mathrm{Tr}\left(H\right)-\epsilon_{0}. \end{array}$$

If the dimension of the ancilla $d_{A}$ is greater or equal to $d_{S}$, this value is attained by using—among others—the classical-classical state

(23) $$\begin{array}{c} \varrho^{SA}=\frac{1}{d_{S}}\sum_{i=1}^{d_{S}}|s_{i}\rangle \langle s_{i}{|}^{S}⊗|a_{i}\rangle \langle a_{i}{|}^{A} \end{array}$$

and the projective measurement with effects $|a_{i}\rangle \langle a_{i}{|}^{A}$, where ${\{\;|a_{i}\rangle }^{A}\}$ (${\{\;|s_{i}\rangle }^{S}\}$) forms an orthogonal basis of *A* (*S*). In the above example, the bound is also achievable with maximally entangled pure states

(24) $$\begin{array}{c} \;|\Psi^{SA}\rangle =\frac{1}{\sqrt{d_{S}}}\sum_{i=1}^{d_{S}}\;|s_{i}\rangle^{S}\;{|a_{i}\rangle }^{A}. \end{array}$$

The maximal daemonic gain is, however, not always achieved using pure states, as the following example shows. Consider the following classical-classical state with a qutrit system and a qubit ancilla

(25) $$\varrho^{SA}=\frac{1}{3}{[|0\rangle \langle 0|}^{S}⊗{\left|0\rangle \langle 0\right|}^{A}+{(|1\rangle \langle 1|}^{S}+|2\rangle \langle 2{|}^{S})⊗|1\rangle \langle 1{|}^{A}].$$

For a Hamiltonian with eigenvalues $\epsilon_{0}\leq \epsilon_{1}\leq \epsilon_{2}$ one easily finds the daemonic gain $\delta W\left(\varrho \right)=\left(\epsilon_{2}-\epsilon_{0}\right)/3$. On the other hand, for any pure state, including maximally entangled states, we have

(26) $$\begin{array}{c} \delta W{(\;|\Psi \rangle }^{SA})\leq \frac{1}{2}\left(\epsilon_{1}-\epsilon_{0}\right), \end{array}$$

since the Schmidt-rank of a pure state on a 3×2 dimensional system is at most 2. For a suitably chosen Hamiltonian, such as $H/\epsilon_{1}=\left|1\rangle \langle 1\right|+\epsilon |2\rangle \langle 2|$, with $\epsilon =\epsilon_{2}/\epsilon_{1}>3/2$, the daemonic gain of $\varrho^{SA}$ [Equation (25)] exceeds the daemonic gain of any pure state of the same system.

## 6. Multipartite Daemonic Ergotropy

In this section we want to investigate a multipartite adaptation of the daemonic ergotropy protocol. Concretely, we consider the situation in which *N* different parties i∈{1,…,N} each own one system $S_{i}$, whose energy they can locally measure using their local Hamiltonian $H^{\left(i\right)}$. The energy of all systems combined will then be evaluated using the Hamiltonian

(27) $$\begin{array}{c} H=\sum_{i=1}^{N}H^{\left(i\right)}. \end{array}$$

Additionally, they can only act on their systems locally, that is using local unitaries. It is only this restriction that makes the protocol multipartite regarding the systems. If arbitrary global unitaries were admitted, this would be equivalent to a situation with a single system consisting of *N* subsystems.

We also take the case into account in which there are *M* ancillas, each owned by a different party k∈{1,…,M}. As we are interested in a genuinely multipartite protocol, each party must resort to local measurements, possibly assisted by classical communication among the parties, yielding outcomes $j_{k}$. After all outcomes are obtained, they are publicly announced and every party *i* performs a unitary on their system $S_{i}$, which may depend on all the outcomes $\vec{j}={\left(j_{k}\right)}_{k=1}^{M}$. We define the multipartite daemonic ergotropy $W_{D}^{\mathrm{mult}}$ to be the maximum amount of energy that can be extracted from a state in this way.

Note that, in spite of the previously imposed restrictions, our notion of multipartite daemonic ergotropy is in fact a generalisation of daemonic ergotropy. This might appear paradoxical at first glance. However, the daemonic ergotropy protocol is equivalent to the protocol of multipartite daemonic ergotropy for one system and one ancilla. This especially includes scenarios in which system and ancilla comprise several subsystems. Studying multipartite daemonic ergotropy is interesting, because it is also applicable to settings, in which the implementation of global measurements and unitaries are unfeasible.

As we are only concerned with local measurements, possibly assisted by classical communication among the parties, all effects of a POVM are of the form

(28) $$\begin{array}{c} E_{\vec{j}}=\overset{M}{\underset{k=1}{⨂}}E_{j_{k}}^{k}. \end{array}$$

We denote the respective conditional states of all systems by $\varrho_{\vec{j}}^{S}={\mathrm{Tr}}_{\left(A_{1}\ldots A_{M}\right)}\left(\varrho^{S_{1}\ldots S_{N}A_{1}\ldots A_{M}}E_{\vec{j}}\right)$ and the conditional state of system $S_{i}$ given a measurement outcome $\vec{j}$ as $\varrho_{\vec{j}}^{i}$. As before, the multipartite daemonic ergotropy can then be expressed in terms of the ergotropy as

(29) $$\begin{array}{c} W_{D}^{\mathrm{mult}}\left(\varrho^{\left\{S_{j}\right\},\left\{A_{k}\right\}},H,E\right)=\sum_{\vec{j}}\sum_{i=1}^{N}W\left(\varrho_{\vec{j}}^{i},H^{\left(i\right)}\right). \end{array}$$

With this result, we can show that contrary to the bipartite case [cf. discussions after Equation (3)] in the multipartite setting projective measurements are in general not optimal for work extraction even for pure states. In order to see this, consider a state $\varrho^{S_{1}A}$ and a purification ${\;|\psi \rangle }^{S_{1}S_{2}A}$, with $\varrho^{S_{1}A}={\mathrm{Tr}}_{S_{2}}(|\psi \rangle \langle \psi {|}^{S_{1}S_{2}A})$. If we now assume that system $S_{2}$ is equipped with a local Hamiltonian $H^{\left(2\right)}=hI$, where *h* is a constant, the multipartite daemonic ergotropy of the purified state is

(30) $$\begin{array}{cc} W_{D}^{\mathrm{mult}}{(\;|\psi \rangle }^{S_{1}S_{2}A},H,E) & =\sum_{\vec{j}}\left[W\left(\varrho_{\vec{j}}^{1},H^{\left(1\right)}\right)+W\left(\varrho_{\vec{j}}^{2},H^{\left(2\right)}\right)\right]\\ & =\sum_{\vec{j}}W\left(\varrho_{\vec{j}}^{1},H^{\left(1\right)}\right)\\ & =W_{D}\left(\varrho^{S_{1}A},H^{\left(1\right)},E\right). \end{array}$$

This result stems from the fact that $H^{\left(2\right)}$ is completely degenerate and the ergotropy vanishes for such Hamiltonians. Thus, also the multipartite daemonic ergotropy of the purification is maximised for the same POVM that also maximises the daemonic ergotropy of $\varrho^{SA}$. Hence, the purification of the qutrit-qubit state stated in Equation (5) is an example for a pure state that requires a POVM to maximise the multipartite daemonic ergotropy. Note, however, that there are also states for which projective measurements are optimal independently of the choice of the Hamiltonian. The first example are states that possess a Schmidt decomposition [28], i.e.,

(31) $$\begin{array}{c} \;|\Psi \rangle =\sum_{i}\sqrt{\lambda_{i}}\;|i_{S_{1}}\ldots i_{S_{n}}i_{A_{1}}\ldots i_{A_{m}}\rangle , \end{array}$$

with $\langle i_{S_{l}}|j_{S_{l}}\rangle =\langle i_{A_{l}}|j_{A_{l}}\rangle =\delta_{ij}\forall i,j,l$. For qubits, these are exactly the states that become separable as soon as one particle is ignored [29]. A famous example is the *m*-partite Greenberger–Horne–Zeilinger (GHZ) state

(32) $$\begin{array}{cc} \;|GHZ\rangle =\frac{1}{\sqrt{2}}( & \;|0_{S_{1}}\ldots 0_{S_{n}}0_{A_{1}}\ldots 0_{A_{m}}\rangle +\;|1_{S_{1}}\ldots 1_{S_{n}}1_{A_{1}}\ldots 1_{A_{m}}\rangle ), \end{array}$$

for which the local projective measurements on |0〉 and |1〉 are optimal, since the conditional state of all systems is a pure product state independently of the outcome and its energy can thus be minimised using local unitaries.

A second class of states for which projective measurements are always optimal are multipartite quantum-classical states

(33) $$\begin{array}{c} \varrho_{S_{1}\ldots S_{n}A}=\sum_{i}\sigma_{i}^{S_{1}\ldots S_{n}}⊗{\left|i\rangle \langle i\right|}^{A}. \end{array}$$

Here, we can recover the proof of Theorem 5 to show that the projective measurement with projectors |i〉〈i| is optimal. The only adaptation to the proof is that the unitaries are now required to be products. Of course this result is still true in the special case when the ancilla is made up of several parties, such that the state can be written as

(34) $$\begin{array}{c} \varrho^{\left\{S_{j}\right\}\ldots \left\{A_{m}\right\}}=\sum_{i}\sigma_{i}^{S_{1}\ldots S_{n}}⊗{\left|i\rangle \langle i\right|}^{A_{1}}⊗{\ldots |i\rangle \langle i|}^{A_{m}}. \end{array}$$

In this case, the optimal measurement consists of the local projective measurements with effects ${\left|i\rangle \langle i\right|}_{A_{k}}$.

## 7. Conclusions

We have significantly extended the concept of daemonic ergotropy to situations involving POVM-based information-gain processes, demonstrating that, in general, one should expect an advantage coming from the use of generalised quantum measurements in ancilla-assisted work-extraction schemes. While the optimal generalised measurements can be identified analytically in some restricted—yet physically relevant—cases, we have proposed an SDP-based see-saw algorithm for their construction. This has led to a number of results shedding light on previously unreported issues linked to daemonic approaches to quantum work extraction: while the interplay between quantum correlations and the features of the optimal measurements appears to be intricate, the structure of entanglement sharing in a multipartite scenario where only local unitaries and POVMs are used turns out to be key in the performance of ancilla-assisted work extraction.

Our work paves the way to a number of interesting developments aimed at exploring further and clarifying the relation between quantum features and work-extraction games in quantum scenarios. On the one hand, it will be very interesting to further compare, quantitatively, the performance of daemonic protocols under optimal PVMs and POVMs to ascertain the extents of the benefits induced by the latter class of measurements against the difficulty of practically implement them. On the other hand, the analysis that we have reported here leaves room to the in-depth assessment of multipartite daemonic gain against the structure of multipartite entanglement aimed at the identification of potentially *optimal* classes of multipartite entangled states, when gauged against their role as a resource in work-extraction schemes.

## Appendix A. POVM Advantage in Qutrit-Qubit Example

We present the state

(A1) $$\begin{array}{c} \varrho^{SA}=\frac{1}{3}\sum_{j=0}^{2}\left|j\rangle \langle j\right|⊗P_{j} \end{array}$$

with

(A2) $$\begin{array}{c} P_{j}=\Pi \left(\frac{2\pi j}{3},0\right) \end{array}$$

and

(A3) $$\Pi \left(\alpha ,\beta \right)=\frac{1}{2}\left\{I+\cos\left(\alpha \right)\sigma_{z}+\sin\left(\alpha \right)\left[\cos\left(\beta \right)\sigma_{x}-\sin\left(\beta \right)\sigma_{y}\right]\right\}$$

as an example in which higher daemonic ergotropy can be achieved with POVMs compared to projective measurements, if a Hamiltonian is chosen suitably. Here, we work out the details and show all necessary calculations explicitely. First, we find the optimal projective measurements. It turns out, that they can be found independently of the chosen Hamiltonian. With this result and bearing in mind that the daemonic gain is invariant under unitary transformations of the Hamiltonian, we can then compute the daemonic ergotropy as a function of the energy spectrum.

Since the ancilla is a qubit, there are only two types of projective measurements: Either, the projective measurement has one outcome that is obtained with certainty, which makes the measurement trivial, or the measurement has two outcomes. In the latter case, the effects are rank one. Therefore, we can compute the maximal daemonic gain for projective measurements by computing it for the measurement $\Pi =\left(\Pi (\alpha ,\beta ),\Pi (\alpha +\pi ,\beta )\right)$ and optimise over the angles α and β afterwards. We have

$$\begin{array}{cc} \varrho^{S}= & \frac{1}{3}(|0\rangle \langle 0|+\left|1\rangle \langle 1\right|+|2\rangle \langle 2|),\\ \varrho_{\alpha }^{S}= & \mathrm{Tr}[\varrho^{SA}\left(I⊗\Pi \left(\alpha ,\beta \right)\right)]\\ = & \frac{1}{3}\{|0\rangle \langle 0|\mathrm{Tr}\left[P_{0}\Pi \left(\alpha ,\beta \right)\right]+|1\rangle \langle 1|\mathrm{Tr}\left[P_{1}\Pi \left(\alpha ,\beta \right)\right]+|2\rangle \langle 2|\mathrm{Tr}\left[P_{2}\Pi \left(\alpha ,\beta \right)\right]\}\\ = & \frac{1}{3}\left[\left|0\rangle \langle 0\right|\frac{1}{2}\left(1+\cos\left(\alpha \right)\right)\right.+\left|1\rangle \langle 1\right|\left(\frac{1}{2}-\frac{1}{4}\cos\left(\alpha \right)+\frac{\sqrt{3}}{4}\sin\left(\alpha \right)\cos\left(\beta \right)\right)\\ + & \left|2\rangle \langle 2\right|\left(\frac{1}{2}-\frac{1}{4}\cos\left(\alpha \right)-\frac{\sqrt{3}}{4}\sin\left(\alpha \right)\cos\left(\beta \right)\right)], \end{array}$$

(A4) $$\begin{array}{cc} \varrho_{\alpha +\pi }^{S}= & \mathrm{Tr}[\varrho_{SA}\left(I⊗\Pi \left(\alpha +\pi ,\beta \right)\right)]\\ = & \frac{1}{3}\left[\left|0\rangle \langle 0\right|\frac{1}{2}\left(1-\cos\left(\alpha \right)\right)\right.+\left|1\rangle \langle 1\right|\left(\frac{1}{2}+\frac{1}{4}\cos\left(\alpha \right)-\frac{\sqrt{3}}{4}\sin\left(\alpha \right)\cos\left(\beta \right)\right)\\ + & \left|2\rangle \langle 2\right|\left(\frac{1}{2}+\frac{1}{4}\cos\left(\alpha \right)+\frac{\sqrt{3}}{4}\sin\left(\alpha \right)\cos\left(\beta \right)\right)]. \end{array}$$

From the definition of ergotropy one can easily see that the ergotropy of the conditional states $\gamma_{\alpha }^{S}$ and $\gamma_{\alpha +\pi }^{S}$ will be maximal for cos(β)=±1. This becomes clear when considering a state

(A5) $$\begin{array}{c} \varrho =a|0\rangle \langle 0|+(b+c)|1\rangle \langle 1|+(b-c)|2\rangle \langle 2|, \end{array}$$

where a,b,c∈R and c≥0. Let the Hamiltonian be

(A6) $$\begin{array}{c} H=\epsilon_{0}|\epsilon_{0}\rangle \langle \epsilon_{0}|+\epsilon_{1}|\epsilon_{1}\rangle \langle \epsilon_{1}|+\epsilon_{2}|\epsilon_{2}\rangle \langle \epsilon_{2}|. \end{array}$$

Then, the ergotropy can without loss of generality be written as

(A7) $$\begin{array}{cc} W & =\mathrm{Tr}\left[\varrho H\right]-\min_{U}\mathrm{Tr}\left[U\varrho U^{†}H\right]\\ & =\mathrm{Tr}\left[\varrho H\right]-[\epsilon_{0}a+\epsilon_{1}\left(b+c\right)+\epsilon_{2}\left(b-c\right)]\\ & =\mathrm{Tr}\left[\varrho H\right]-[a\epsilon_{0}+b\left(\epsilon_{1}+\epsilon_{2}\right)+c\left(\epsilon_{1}-\epsilon_{2}\right)], \end{array}$$

where the energy eigenvalues are ordered such that the minimum is achieved. Consequently, we get $\epsilon_{1}\leq \epsilon_{2}$ since (b+c)≥(b−c). Therefore, *W* increases with *c* and we can set β=0 in the above calculation. Exploiting addition theorems, we can now write

$$\begin{array}{cc} \varrho_{\alpha }^{S}= & \frac{1}{6}\left[|0\rangle \langle 0|\left[1+\cos\left(\alpha \right)\right]+\left|1\rangle \langle 1\right|\left(1+\cos\left(\alpha -\frac{2\pi }{3}\right)\right)\right.+\left|2\rangle \langle 2\right|\left(1+\cos\left(\alpha +\frac{2\pi }{3}\right)\right)]\\ \varrho_{\alpha +\pi }^{S}= & \frac{1}{6}\left[|0\rangle \langle 0|\left(1-\cos\alpha \right)+\left|1\rangle \langle 1\right|\left(1-\cos\left(\alpha -\frac{2\pi }{3}\right)\right)\right.+\left|2\rangle \langle 2\right|\left(1-\cos\left(\alpha +\frac{2\pi }{3}\right)\right)]. \end{array}$$

As one can easily see, an optimal value of α is not unique, as shifting its value by $\frac{2\pi }{3}$ can be compensated by relabeling the states, which does not affect the daemonic gain. We now aim to find the optimal α in the interval $\left[-\frac{\pi }{3},\frac{\pi }{3}\right)$. When calculating the ergotropy of the conditional states we need to know the ordering of their eigenvalues

(A8) $$\begin{array}{c} \alpha \in \left[-\frac{\pi }{3},0\right)\Rightarrow \cos\left(\alpha \right)\geq \cos\left(\alpha +\frac{2\pi }{3}\right)\geq \cos\left(\alpha -\frac{2\pi }{3}\right)\\ \alpha \in \left(0,\frac{\pi }{3}\right)\Rightarrow \cos\left(\alpha \right)\geq \cos\left(\alpha -\frac{2\pi }{3}\right)\geq \cos\left(\alpha +\frac{2\pi }{3}\right) \end{array}$$

In the following calculation, the upper sign will refer to the negative and the lower sign will refer to the positive interval

(A9) $$\begin{array}{ccc} \delta W(\varrho_{SA},H,\Pi ) & = & W_{D}\left(\varrho_{SA},H,\Pi \right)-W\left(\varrho_{S},H\right)\\ & = & \mathrm{Tr}\left[\varrho_{S}H\right]-\min_{\Pi }\sum_{k}\mathrm{Tr}\left[\varrho_{S}A\left(U_{k}^{†}HU_{k}⊗\Pi_{k}\right)\right]-\left[\mathrm{Tr}\left[\varrho_{S}H\right]-\min_{U}\mathrm{Tr}\left[\varrho_{S}U^{†}HU\right]\right]\\ & = & \min_{U}\mathrm{Tr}\left[\varrho_{S}U^{†}HU\right]-\min_{\Pi }\sum_{k}\mathrm{Tr}\left[\varrho_{S}A\left(U_{k}^{†}HU_{k}⊗\Pi_{k}\right)\right]\\ & = & \max_{\alpha }\{\frac{1}{3}\left(\epsilon_{0}+\epsilon_{1}+\epsilon_{2}\right)-\frac{1}{6}\left(\epsilon_{0}\left[1+\cos\left(\alpha \right)\right]\right)+\epsilon_{1}\left(1+\cos\left(\alpha \pm \frac{2\pi }{3}\right)\right)\\ & & +\epsilon_{2}\left(1+\cos\left(\alpha \mp \frac{2\pi }{3}\right)\right)+\epsilon_{0}\left(1-\cos\left(\alpha \mp \frac{2\pi }{3}\right)\right)\\ & & +\epsilon_{1}\left(1-\cos\left(\alpha \pm \frac{2\pi }{3}\right)\right)+\epsilon_{2}\left(1-\cos\alpha \right)\}\\ & = & \frac{1}{6}\left(\epsilon_{2}-\epsilon_{0}\right)\max_{\alpha }\left(\cos\left(\alpha \right)-\cos\left(\alpha \mp \frac{2\pi }{3}\right)\right)\\ & = & \frac{\epsilon_{2}-\epsilon_{0}}{2\sqrt{3}}. \end{array}$$

Now, that we computed the maximal daemonic gain for projective measurements, we compare this with the daemonic gain that can be achieved by using the POVM *M*, consisting of the effects $\frac{2}{3}P_{i}$, as defined in Equation (A2). In this case, the conditional states are

(A10) $$\begin{array}{c} \gamma_{P_{0}}^{S}=\frac{2}{9}\left(\left|0\rangle \langle 0\right|+\frac{1}{4}\left|1\rangle \langle 1\right|+\frac{1}{4}\left|2\rangle \langle 2\right|\right),\\ \gamma_{P_{1}}^{S}=\frac{2}{9}\left(\frac{1}{4}\left|0\rangle \langle 0\right|+\left|1\rangle \langle 1\right|+\frac{1}{4}\left|2\rangle \langle 2\right|\right),\\ \gamma_{P_{2}}^{S}=\frac{2}{9}\left(\frac{1}{4}\left|0\rangle \langle 0\right|+\frac{1}{4}\left|1\rangle \langle 1\right|+\left|2\rangle \langle 2\right|\right). \end{array}$$

Given the conditional states, we can now compute the daemonic gain as

(A11) $$\begin{array}{cc} \delta W= & \epsilon_{0}\left(\frac{1}{3}-\frac{2}{3}\right)+\epsilon_{1}\left(\frac{1}{3}-\frac{1}{6}\right)+\epsilon_{2}\left(\frac{1}{3}-\frac{1}{6}\right)\\ = & -\frac{1}{3}\epsilon_{0}+\frac{1}{6}\left(\epsilon_{1}+\epsilon_{2}\right). \end{array}$$

Choosing the Hamiltonian $H=|\epsilon_{1}\rangle \langle \epsilon_{1}\left|+\right|\epsilon_{2}\rangle \langle \epsilon_{2}|$ provides an example where the maximal daemonic gain can not be achieved by using projective measurements because

(A12) $$\begin{array}{c} \delta W_{\mathrm{proj}}=\frac{1}{2\sqrt{3}}<\delta W_{M}=\frac{1}{3}. \end{array}$$

## Appendix B. Non-Optimal Convergence of the See-Saw Algorithm

In the following, we construct a case in which Algorithm 1 will yield a sequence of values for the daemonic ergotropy that does not converge against the maximal daemonic ergotropy. Consider a state $\varrho^{SA}$ on a system *S* with a Hamiltonian *H* and a *d*-dimensional ancilla *A*, such that the optimal measurements are rank-one projective measurements as long as only *d*-outcome measurements are considered. Then, there exists an initialisation of Algorithm 1, such that the sequence of daemonic ergotropies generated by the algorithm limits in the maximal daemonic ergotropy for *d*-outcome measurements. In order to see this, consider a measurement Π that is optimal among *d*-outcome measurements. For the effects $\left\{\Pi_{1},\ldots ,\Pi_{d}\right\}$ one finds *d* optimal unitaries $\left\{V_{1},\ldots ,V_{d}\right\}$. We now initialise the algorithm for $d^{2}$ outcomes in the following way

(A13) $$\begin{array}{cc} U_{i} & =V_{i},\;\;\;\;\;i=1,\ldots ,d-1\\ U_{i} & =V_{d},\;i=d,\ldots ,d^{2}. \end{array}$$

This implies $\tau_{d}=\tau_{d+1}=\ldots =\tau_{d^{2}}$, where $\tau_{i}\;=\;{\mathrm{Tr}}_{S}\left(\varrho^{SA}U_{i}^{†}HU_{i}\right)$. Hence, the objective of step 2 of the algorithm simplifies to

(A14) $$\begin{array}{c} \min_{M}\sum_{i=1}^{d^{2}}\mathrm{Tr}\left(\tau_{i}M_{i}\right)=\min_{M}\left[\sum_{i=1}^{d-1}\mathrm{Tr}\left(\tau_{i}M_{i}\right)+\mathrm{Tr}\left(\tau_{d}\sum_{j=d}^{d^{2}}M_{j}\right)\right]. \end{array}$$

The value of this expression thus depends on *d* effects $M_{1},\ldots ,M_{d-1},\sum_{j=d}^{d^{2}}M_{j}$. In this case, the minimum can by assumption only be achieved if the effects are all rank-one. This implies that the first d−1 effects are orthogonal rank-one projectors and the remaining effects are rank-one operators on the remaining one-dimensional subspace and sum up to a rank-one projector. Thus, the algorithm again finds a *d*-outcome rank-one projective measurement that is optimal among *d*-outcome measurements. The case that was discussed above is of practical relevance, as we have observed in numerical experiments that randomly initialised unitaries may converge against the configuration stated in Equation (A13).

The example discussed in Appendix A meets the requirement that all optimal two-outcome measurements are rank-one projective measurements. The optimal projective measurements are calculated in Appendix A. Any two outcome measurement in two dimensions with rank-two effects can be considered as a mixture of a rank-one projective measurement with white noise. The only case, in which white noise will not decrease the daemonic ergotropy is, if the conditional states $\gamma_{i}^{S}$ [Equation (A8)] are simultaneously diagonalisable by the same diagonalising unitary and with the same ordering of eigenvalues in diagonal form. This is however not the case, since both states are already diagonal but the eigenvalues are not in the same order.

In the same example, the maximum daemonic ergotropy cannot be achieved with *d*-outcome measurements.

## Appendix C. Proof of Theorem 5

In this Appendix we provide a complete proof of the statement made in Theorem 5, which we repeat here again for easiness of reading. For a quantum-classical state, that is a state that can be cast in the form

(A15) $$\begin{array}{c} \varrho_{qc}^{SA}=\sum_{j}\sigma_{j}^{S}⊗{\left|j\rangle \langle j\right|}^{A} \end{array}$$

with a set of orthonormal vectors ${\{\;|j\rangle }^{A}\}$ and unnormalised states $\sigma_{j}^{S}$ the following theorem holds.

**Theorem** **A1.**
*For a quantum-classical state $\varrho_{qc}^{SA}$, the maximum daemonic ergotropy is*

(A16) $$\begin{array}{c} \max_{M}W_{D}\left(\varrho^{SA},H,M\right)=\sum_{j}W\left(\sigma_{j}^{S},H\right). \end{array}$$

*This value is achieved by performing the projective measurements $P_{j}={\left|j\rangle \langle j\right|}^{A}$ on the ancilla A.*

**Proof.** The first claim follows directly from the second claim using Equation (3). Therefore, we prove the second claim by showing that the daemonic gain achieved through any POVM *E* with effects $E_{i}$ and an arbitrary number of outcomes *N* has an upper bound given by the value corresponding to the use of projective measurements. We start by computing the conditional states

(A17) $$\begin{array}{c} \gamma_{k}^{S}={\mathrm{Tr}}_{A}\left[\varrho^{SA}\left(I⊗E_{k}\right)\right]=\sum_{j=1}^{d}\sigma_{j}^{S}\langle j|\;E_{k}\;|j\rangle . \end{array}$$

It can be easily seen that post-processing can never increase the daemonic ergotropy. This allows us to assume, without loss of generality, that all effects are rank-one and use Naimark’s extension theorem [30] to write

(A18) $$\gamma_{k}^{S}=\sum_{j=1}^{N}\sigma_{j}^{S}{\left|\langle j|\phi_{k}\rangle \right|}^{2},$$

where $(|\phi_{k}\rangle {\langle \phi_{k}|)}_{k=1}^{N}$ is the Naimark extension of the operators $E_{k}$ on the extended ancilla space. Then, $(\;|\phi_{k}\rangle {))}_{k=1}^{N}$ is an orthonormal basis in the extended ancilla space. We also extend ${(\;|j\rangle )}_{j=1}^{d}$, so ${(\;|j\rangle )}_{j=1}^{N}$ is another orthonormal basis in the extended ancilla space and set $\sigma_{j}^{S}=0,\;\forall j>d$. We can now interpret $|\langle j|\phi_{k}\rangle {|}^{2}$ as entries of a doubly stochastic matrix and apply the Birkhoff-von Neumann theorem [31], which allows us to express this doubly stochastic matrix as a convex combination of permutation matrices $\pi^{\left(n\right)}={\left(\pi_{jk}^{\left(n\right)}\right)}_{jk}$. This yields

(A19) $$\gamma_{k}^{S}=\sum_{j=1}^{N}\sigma_{j}\sum_{n}p_{n}\pi_{jk}^{\left(n\right)}$$

with probabilities $p_{n}$. We insert this result into the formula of the daemonic ergotropy

(A20) $$\begin{array}{c} W_{D}\left(\varrho^{SA},H,M\right)=\mathrm{Tr}\left(\varrho^{S}H\right)-\sum_{k}\min_{U_{k}}\mathrm{Tr}\left(U_{k}\gamma_{k}^{S}U_{k}^{†}H\right). \end{array}$$

As we are interested in the optimal measurement, our only concern is the second term

(A21) $$\begin{array}{cc} & \sum_{k=1}^{N}\min_{U}\mathrm{Tr}\left(U\gamma_{k}^{S}U^{†}H\right)\\ = & \sum_{k=1}^{N}\min_{U}\mathrm{Tr}\left(U\sum_{j=1}^{N}\sigma_{j}\sum_{n}p_{n}\pi_{jk}^{\left(n\right)}U^{†}H\right)\\ \geq & \sum_{k,j,n}p_{n}\pi_{jk}^{\left(n\right)}\min_{U}\mathrm{Tr}\left(U\sigma_{j}U^{†}H\right)\\ = & \sum_{n}p_{n}\sum_{j}\min_{U}\mathrm{Tr}\left(U\sigma_{j}U^{†}H\right)\sum_{k}\pi_{jk}^{\left(n\right)}\\ = & \sum_{j}\min_{U}\mathrm{Tr}\left(U\sigma_{j}U^{†}H\right), \end{array}$$

which is bounded from below by the value that is achieved for the projective measurement $P_{j}=\left|j\rangle \langle j\right|$, as stated above. □
